# Relationships between strength and endurance parameters and air depletion rates in professional firefighters

**DOI:** 10.1038/srep44590

**Published:** 2017-03-17

**Authors:** Stephanie Windisch, Wolfgang Seiberl, Ansgar Schwirtz, Daniel Hahn

**Affiliations:** 1 Department of Biomechanics in Sports, Technical University of Munich, Germany; 2 Human Movement Science, Ruhr-University Bochum, Germany; 3 School of Human Movement and Nutrition Sciences, University of Queensland, Australia.

## Abstract

The aim of this study was to quantify the physical demands of a simulated firefighting circuit and to establish the relationship between job performance and endurance and strength fitness measurements. On four separate days 41 professional firefighters (39 ± 9 yr, 179.6 ± 2.3 cm, 84.4 ± 9.2 kg, BMI 26.1 ± 2.8 kg/m^2^) performed treadmill testing, fitness testing (strength, balance and flexibility) and a simulated firefighting exercise. The firefighting exercise included ladder climbing (20 m), treadmill walking (200 m), pulling a wire rope hoist (15 times) and crawling an orientation section (50 m). Firefighting performance during the simulated exercise was evaluated by a simple time-strain-air depletion model (TSA) taking the sum of z-transformed parameters of time to finish the exercise, strain in terms of mean heart rate, and air depletion from the breathing apparatus. Multiple regression analysis based on the TSA-model served for the identification of the physiological determinants most relevant for professional firefighting. Three main factors with great influence on firefighting performance were identified (70.1% of total explained variance): VO_2peak_, the time firefighter exercised below their individual ventilatory threshold and mean breathing frequency. Based on the identified main factors influencing firefighting performance we recommend a periodic preventive health screening for incumbents to monitor peak VO_2_ and individual ventilatory threshold.

Many studies proved evidence for the high physical strain induced by firefighting activity^1^,^2^,^3^,^4^,^5^,^6^,^7^,^8^. The studies revealed that firefighters showed physiological responses of 80% of heart rate maximum (HR_max_) on average with a range from 60–90% HR_max_ (e.g. refs 3, 5, 7 and 9). These previous studies used physically demanding simulated firefighting tasks to characterize the physiological responses during such activities. Research focused on oxygen uptake (VO_2_) or heart rates (HR), and quantified push- and pull forces in order to relate the outcome to aerobic fitness and muscular strength with the main goal to establish the relationship between job demands and fitness parameters^10^,^11^,^12^,^13^. VO_2peak_, hand grip strength, number of push-ups and pull-ups completed were some of the most common found fitness variables to be important for firefighters^9^,^13^,^14^. However, the physical strain induced by firefighting can be a limiting factor for firefighting performance. High strain, e.g. working with anaerobic metabolism over a long period of time, requires a high fitness level to maintain operating speed.

The primary focus when assessing firefighting performance in previous research lay on completion time of the simulated firefighting exercises as the performance determining parameter^11^,^15^,^16^. Therefore, previous studies showed positive correlations between completion time and fitness variables^11^,^16^,^17^. Other researchers predicted performance time by multiple regression^2^,^9^,^12^,^14^,^15^. Doubtless, time is a critical parameter for firefighters. When they arrive at an emergency scene, they have to work as fast as possible in order to prevent the spread of burning fires, destruction of property as well as to save lives of victims. However, apart from time there can be other limiting factors such as compressed air depletion from the self-containing breathing apparatus (SCBA). This air cylinder has a nominal capacity of recirculating compressed air for approximately 30 minutes, however, previous observations showed that the capacity of the SCBA was exceeded before the end of the exercise^18^. The lower air depletion, the longer a firefighter can work at an emergency scene and prolong interventions requiring air cylinder use. Aside from two studies that showed high rates of air consumption during simulated firefighting, air depletion from the SCBA has hardly been researched yet^19^,^20^. Moreover, the relationship between fitness variables and air depletion has not been established yet.

Together with well-researched parameters of completion time and physical strain, we propose that air depletion from the SCBA can provide additional, extremely valuable information. To our knowledge, there is no study researching firefighting performance as a combination of these three parameters. For this study, we therefore defined a simple formula to quantify the demands of the simulated firefighting exercise adding time needed for the exercise, heart rate and air depletion from SCBA. The aim of our study was 2-fold: (1) Quantification of the physical demands of a simulated firefighting exercise by the simple formula taking into account completion time of the exercise, heart rate and air depletion rate. (2) Establishment of the relationship between firefighting performance and highly standardized fitness measurements in order to identify the most relevant physical and physiological attributes to fulfill the job demands of a professional firefighter. From this approach we expected to characterize firefighting more in detail. We hypothesized that firefighters with lower air depletion from the SCBA, fast completion time and lower physical strain during the simulated firefighting exercise possess a higher fitness level.

## Materials and Methods

### Subjects

Forty-one male career firefighters (39 ± 9 yr, 179.6 ± 2.3 cm, 84.4 ± 9.2 kg, BMI 26.1 ± 2.8 kg/m^2^) of the Munich Airport volunteered to participate in the research. Full written and verbal details about the study were provided. Informed written consent was obtained from all participants prior to testing. The ethic statement for this study was approved by the Dean of the Faculty of Sports and Health Sciences of the Technical University of Munich. All tests were conducted according to the Declaration of Helsinki. All participants were in possess of a valid G26.3 medical examination for operational fitness, a mandatory periodically medical health check for firefighters in Germany.

### Material and methods

The tests were conducted on four days each separated by at least 4 days in the following order: *Day 1* - Test of VO_2peak_ during maximal treadmill running and anthropometric evaluation. *Day 2* - Flexibility, balance, muscular strength and muscular endurance testing. *Day 3* - Respiratory protection exercise (REPE_standard_) with SCBA. *Day 4*: Respiratory protection exercise (REPE_spirometry_) with a spirometry mask.

All subjects wore functional sportswear and -shoes during the VO_2_ peak testing, muscular strength and endurance testing.

#### Anthropometric evaluation

Body mass (kg) was recorded with the nearest 0.1 kg on a scale with shoes removed. Body height was measured by a tapeline with the nearest 0.1 cm of the maximum distance from the floor to the vertex of the head with shoes removed. Body Mass Index (BMI) was calculated by the following formula: Bodyweight in kilograms divided by height in meters squared (kg/m^2^).

#### VO_2_ peak testing

Minute ventilation (V_E_) and gas exchange (oxygen consumption - VO_2_, carbon dioxide output - VCO_2_, respiratory exchange ratio - RER) were measured breath-by-breath with the Cortex Metamax 3B (Cortex Biophysics GmbH, Germany). An incremental exercise test based on the Ellestad Protocol^21^ was conducted on a motorized treadmill (Life Fitness, Integrity Series, Germany) to determine peak oxygen uptake (VO_2peak_), minute ventilation (V_E)_ and heart rate maximum (HR_max_). The test was terminated when subjects reached volitional fatigue and were not able to continue running. VO_2peak_ and HR_max_ were taken as the highest 30s-average during the final minute of the test. In addition, two thresholds were determined based on the test: ventilatory threshold 1 (VT1) and respiratory compensation point (RCP). The VT1 was determined from the V-slope method^22^ in combination with the break point of the ventilatory equivalent for O_2_ against VO_2_^23^. The RCP was identified by the break points of the ventilatory equivalent for CO_2_ and the end tidal CO_2_ concentration against VO_2_^23^. These two thresholds were then used to establish three physiological intensity zones that correspond to the heart rates at the following exercise intensities: Zone 1, 2 and 3 were represented by the percentage of time subjects experienced HR below VT1 (Zone 1), HR between VT1 and RCP (Zone 2) and HR above RCP (Zone 3), respectively.

#### Flexibility, balance, muscular strength and endurance testing

A description of standardized fitness tests can be found in Table 1. All tests were completed sequentially with a break of at least 4 minutes between each test. A standardized warm up of 20 minutes on a cross-trainer (Life Fitness, Integrity Series, Germany) preceded the tests.

#### Simulated firefighting exercise test protocol

The simulated firefighting exercise was completed twice by each subject. One trial was with wearing full gear and the second trial was with full gear, but without the facemask, and wearing a portable metabolic measurement system.

Respiratory Protection Exercise Standard (REPE_standard_): This exercise is a standardized, mandatory and periodically performed ability test for professional German firefighters. The test was conducted as prescribed by German firefighting test regulations^24^. Subjects were tested in a purpose-built practice area, wearing full personal protection gear (clothing, helmet, gloves, belt, facial mask, boots) and SCBA. The SCBA cylinders were filled with 300 bar according to the standard protocol for the fire services. The weight of the protection gear and SCBA was approximately 22 kg. The tasks included *ladder climb* (20 m), a 200 m *treadmill walk*, pulling a wire rope *hoist* (15 times) and *crawling* a 50 m *orientation section* in the dark with bottlenecks and a narrow tunnel. Subjects were instructed to perform the REPE safely and as fast as possible but in a pace similar to the work at a real fire emergency scene. The tasks were performed in succession without interruption but with individually chosen pace and possible breaks in case of exhaustion. During the REPE_standard_, heart rate was measured continuously (Polar, Finland) and ratings of perceived exertion^25^ as well as air depletion from the SCBA were taken at the end of the exercise. Individual task time and total performance time were recorded.

Respiratory Protection Exercise with Spirometry (REPE_spirometry_): The exercise protocol for the REPE_spirometry_ was identical with the testing of the REPE_standard_ but included spirometric measurements. This measurement provided additional information in terms of respiratory variables during firefighting. The standard facial mask of the SCBA was replaced by the mobile spirometry mask of the Cortex Metamax device to measure VO_2_, VCO_2_, VE, RER and ventilatory equivalents (VE/VO_2_; VE/VCO_2_). These variables were measured breath-by-breath and were then used to define the metabolic demands of the REPE. Subjects still wore the SCBA (without facial mask) to simulate the weight of their equipment.

#### Firefighting performance formula

We defined a simple formula to quantify the demands of the simulated firefighting exercise adding time needed for the exercise, mean heart rate during exercise expressed as percentage of the treadmill determined HR_max_ and air depletion from SCBA. We included the three variables in our formula because we defined optimal firefighting performance due to three important key aspects: (1) How much time do firefighters need to complete a given simulated firefighting exercise ? (2) What are their physiological responses to the chosen operation speed? (3) How much air do they consume from their SCBA due to operation speed and work intensity? We defined this as the time-strain-air depletion (TSA) formula resulting in a TSA score:


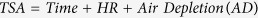


As the impact of every single factor on overall TSA-score is not clear at the moment, we used z-transformations to prevent different weighting of the parameters due to their different absolute values and normal distributions. The resulting z-scores allow us to compare and sum up the three parameters resulting in the TSA-score. As this score is based on the function of a z-score, the TSA-score indicates the resultant firefighting performance in relation to the sample mean, with the distance measured in standard deviations. A TSA-score of 0 represents the average. We ranked performers according to their TSA-scores into 5 categories based on standard deviations: “*Outstanding*” (TSA < −2), “*Above Average*” (TSA −1 to −2), “*Average*” (TSA −0.99 to +0.99), “*Below Average*” (TSA 1 to 2), and “*Poor*” (TSA > 2). Individual performance scores for the TSA should be kept at a minimum achieved through fast completion time, low heart rate as well as low air depletion during the exercise.

### Data analyses

Statistical calculations were carried out with SPSS version 23.0 (IBM Corporation, USA). Descriptive statistics (means, standard deviations (SD)) were calculated to define subjects with respect to physical characteristics and performance in the tests. For legpress, handgrip and one-leg standing, data were taken as the average of left and right. Data were assumed to be normally distributed if the Shapiro-Wilk’s test was >0.05. As all data was normally distributed, parametric tests were consequently carried out. The alpha level was set to 0.05. A paired t-test was calculated to show up differences between the two firefighting exercises REPE_standard_ and REPE_spirometry_. Reference values according to Cohen^26^ were used to interpret the correlations. Values from 0.10–0.29 were considered ‘small’, 0.30–0.49 ‘moderate’ and ≥0.50 ‘strong’. The combination of physical characteristics that best predict TSA was determined by multiple regression (Enter Method). The combination of variables that resulted in the highest explained variance that predicted the largest portion of the variance was then selected.

## Results

### Aerobic fitness, muscular strength, flexibility and balance testing

Table 2 provides an overview over the results of treadmill, muscular strength, flexibility and balance testing.

### Physiological demands of the REPE_standard_

Total exercise time averaged 801 ± 129 s (13.4 ± 2.2 min). The time required for completion of each of the four tasks during the REPE_standard_ was 85 ± 15 s (1.4 ± 0.2 min) for *ladder climb*, 141 ± 13 s (2.3 ± 0.2 min) for *treadmill walking*, 35 ± 8 s (0.6 ± 0.1 min) for *hoist* and 412 ± 96 s (6.9 ± 1.6 min) for *orientation section crawling*. Mean heart rate of the REPE_standard_ was 143.2 ± 12.1 beats per minute (bpm), which corresponded to 79.2 ± 6.6% of maximum heart rate (HR_max_) determined on the treadmill. Mean HR values for *ladder climb* were 81 ± 7.4% of HR_max_ and for *orientation section crawling* 81 ± 6.7% of HR_max_. *Hoist* averaged 78.8 ± 5.1% of HR_max_ and *treadmill walking* 75.4 ± 7.8% of HR_max_.

Subjects spent 21.3 ± 24.3% of total exercise time in Zone 1, 69.9 ± 25.1% of time in Zone 2 and 8.8 ± 17.3% in Zone 3. Mean air depletion from the air cylinder averaged 161.7 ± 28.7 bar. In the first part of the REPE_standard_ (*ladder climb, treadmill walk* and *hoist*), mean air depletion was 85.6 ± 16.8 bar which corresponded to 28.6 ± 5.6% of the capacity of a nominal 30 min-cylinder. In the second part, the *orientation section crawling*, air depletion ended up in 76.3 ± 19.1 bar (25.5 ± 6.3%). In total subjects consumed 54.1 ± 9.9% of the capacity of a nominal 30 min-cylinder.

### Respiratory demands of the REPE_spirometry_

**Figure 1:**
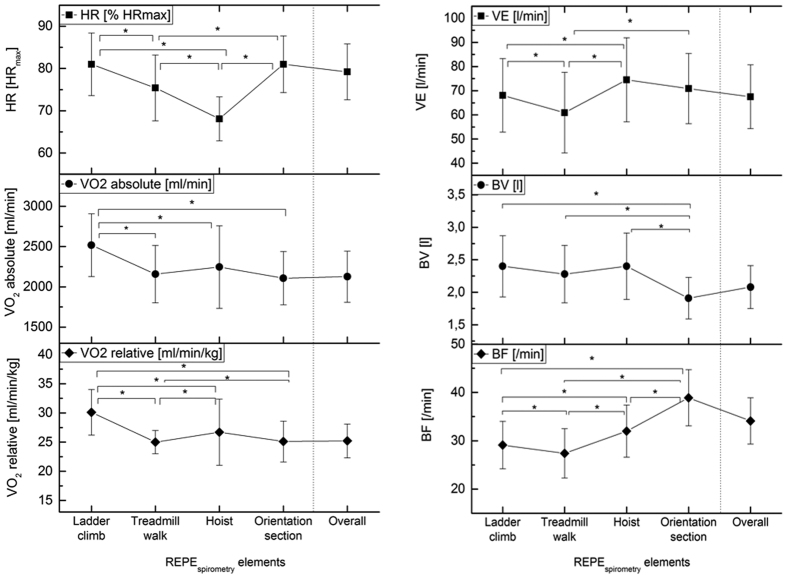
Physiological responses during the REPE: Heart rate (HR), peak oxygen uptake absolute (VO_2peak_ absolute) and relative (VO_2peak_ relative), minute ventilation (VE), breathing volume (BV) and breathing frequency (BF) during ladder climb, treadmill walk, hoist, orientation section and the overall exercise. Data are shown as means ± standard deviations (SD). *Significant difference between tasks (P < 0.05).

Mean heart rate during the REPE_spirometry_ was 144.3 ± 12.7 bpm corresponding to 79.8 ± 7.3% of HR_max_. Exercise total time of the REPE_spirometry_ averaged 797 ± 122 s (13.3 ± 2.0 min). Mean heart rates (*p* = 0.433) and exercise total time (*p* = 0.858) showed no significant differences between REPE_standard_ and REPE_spirometry_. The mean oxygen consumption for the whole REPE_spirometry_ exercise was 2.13 ± 0.32 l/min. Among the different exercise elements, *ladder climb* required the highest absolute oxygen uptake (2.51 ± 0.39 l/min). Corrected for body mass, mean VO_2_ was 25 ± 3 ml/min/kg across the whole exercise, 30 ± 4 ml/min/kg during *ladder climb*, 27 ± 6 ml/min/kg during *hoist* and 26 ± 6 ml/min/kg both during *treadmill walk* and the *orientation section crawling.* The two most demanding tasks required 38 ± 6 ml/min/kg over 20 seconds *during orientation section crawling* and 38 ± 5 ml/min/kg at the *ladder climb* (Fig. 1).

Mean minute ventilation during the whole exercise was 67.5 ± 13.1 l/min. *Hoist* showed the highest mean minute ventilation rate (74.5 ± 17.4 l/min), followed by the *orientation section* (70.9 ± 14.5 l/min) and the *ladder climb* (60.9 ± 16.7 l/min). Mean breathing volume values during the exercise were 2.08 ± 0.33 l. *Ladder climb* and *hoist* required breathing volumes of 2.4 l, the *treadmill walk* 2.28 ± 0.45 and the *orientation section* 1.9 ± 0.33 l. The mean breathing frequency was registered with 34.1 ± 4.8 breaths per minute. The *orientation section* required the highest number of breaths per minute (38.9 ± 5.8), followed by *hoist* (32.0 ± 5.5), *ladder climb* 29.1 ± 4.9) and *treadmill walk* (27.4 ± 5.1) (see Fig. 1). Furthermore, respiratory exchange ratio (RER) averaged 1.08 ± 0.08 across the total exercise. 36.0 ± 21.7% of total exercise time subjects had a RER <1.0 and 64.0 ± 21.7% of time a RER ≥1.0.

### Relationship between TSA-score and fitness characteristics

**Figure 2:**
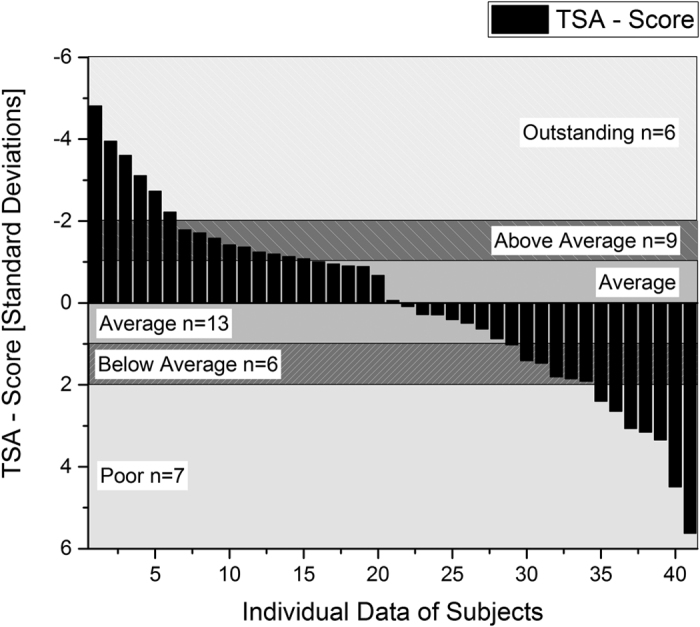
Individual TSA-Scores of all 41 subjects classified into *Outstanding, Above Average, Average, Below Average* and *Poor*.

**Figure 3:**
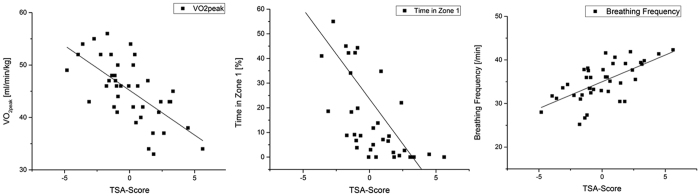
Relationship between the three main performance predictors and TSA-scores identified by multiple regression: relative VO_2peak_ (*left*), time in Zone 1 (*middle*) and breathing frequency (*right*).

Thirteen firefighters obtained a TSA-score of −0.99 to +0.99 (average), 9 firefighters a TSA-score between −1 and −2 (above average) and 6 firefighters a score smaller than −2 (outstanding). Furthermore, 6 firefighters obtained a score between 1 and 2 (below average) and 7 subjects a TSA-score of more than 2 (poor) (Fig. 2). As there was no significant difference between mean HR and mean completion time of REPE_standard_ and REPE_spirometry_, we assumed that strain and duration of both exercises were comparable. Therefore, we used all REPE_standard_ and REPE_spirometry_ variables in addition to variables from treadmill and muscular strength, flexibility and balance testing to find the most predictive parameters for firefighting by multiple regression. Based on our performance model (Table 3), multiple regression identified three main factors that show a great influence on optimal firefighting performance in terms of the TSA-score (70.1% of total explained variance): relative VO_2peak_ from maximum treadmill testing, mean breathing frequency and the percentage of time spent in Zone 1 during REPE_standard_. Figure 3 shows the relationship of all three parameters to TSA-score. To better understand the characteristics of firefighters with respect to different TSA-scores, Table 4 shows TSA-parameters, the main identified variables by regression and additional variables for all categories of performers. It can be noted that outstanding performers had significantly higher VO_2peak_ (*p* = 0*.001*) and significantly lower mean heart rates during REPE (*p* = 0.001) while completing the exercise faster (p = 0.001) compared to *average, below average* and *poor* performers. The differences of VO_2peak_-levels and time spent in zone 1 of different TSA-performers are highlighted in Table 4. The poorest performers also showed an increased perceived exertion when rating the BORG scale after the exercise. Furthermore, the outstanding performers were the only subjects performing the REPE parcours without spending any time in Zone 3 and showing the highest fraction of time spent in Zone 1.

## Discussion

The results of this research describe, for the first time, firefighting performance as a combination of operating speed (time to complete the circuit), physical strain and air depletion during a simulated firefighting exercise.

Firefighting is a physically demanding occupation. Several authors offered evidence for high physiological responses above 80% of peak relative oxygen uptake (VO_2_) during the completion of simulated task circuits^1^,^5^,^7^. Others reported values between 47% and 80% of relative peak oxygen uptake^4^,^8^,^15^,^20^. In our study, relative VO_2_ averaged at 56% of VO_2peak_ across the exercise, which was towards the lower end of the range of average values reported from other studies. The values reported for single firefighting tasks within circuits varied from 23 ml/min/kg for boundary cooling^1^ to 44 ml/min/kg for tower stair climbing^27^. Literature indicates stair climbing^6^,^27^ and victim rescue^28^ to be the most arduous tasks, requiring a VO_2_ of 38–43 ml/min/kg over 20 seconds. These findings are comparable to our values determined for *ladder climb* and *orientation section crawling*, although measured VO_2_ rather represent the lower end of the reported ranges. During the REPE circuit, HR averaged at 79.2 ± 6.6% of HR_max_ determined on the treadmill. These findings were consistent with values reported from other studies ranging from 61%^29^ to 95%^4^ of HR_max_. The most common physiological responses in terms of mean HR during the firefighting exercises averaged between 80% and 90% of HR_max_^3^,^7^,^9^. However, in our study, we analyzed not only mean heart rate but also the time spent in the three defined physiological intensity zones. These zones indicate the contribution of different energy sources to total exercise performance and provide more detailed information on cardiovascular load during firefighting. Subjects spent most time of their exercise time in Zone 2, the aerobic-anaerobic metabolic transition zone (69% of time), whereas the aerobic fraction (Zone 1) represented approximately 22% of exercise time. The smallest fraction (9% of time) was Zone 3 representing an anaerobic metabolism and indicating the onset of hyperventilation. A high fraction of time in Zone 3 will lead to subject’s rapid fatigue, whereas a high fraction of time in Zone 1 confirms a subject’s good aerobic metabolism^30^. To our knowledge, only one other study analyzed the three physiological intensity zones in the same way and found a distribution of energy metabolism of approximately 84% Z1, 12% Z2 and 2% Z3^31^. These results show important differences to our findings, however, it should be noted that the mentioned study investigated prolonged (>120 min) wildland firefighting and exercise time was almost 10 times longer than the simulated firefighting tests we investigated.

Subjects showed a RER ≥ 1.0 during 64% of total exercise time indicating a major contribution of anaerobic energy due to more CO_2_ being produced than O_2_ consumed. An increase of RER above 1.0 would only be expected, if VO_2_ exceeded 89% VO_2peak_^32^. During the maximum treadmill testing in our study, we established an average RER of 1.08 not before subjects ran at an intensity of 90% to 97% of VO_2peak_. However, during the firefighting exercise, subjects reached an average RER 1.08 already at an intensity of 56% of VO_2peak_. These observed RERs were out of line with RERs found during other moderate activities between 50 and 60% VO_2peak_. For example, Davis *et al*.^33^ reported average RER values of 0.85 during treadmill running at exercise intensities of 58% VO_2peak_. Although the mean values for VO_2_ were relatively low in our study, RER averaged 1.08 across the total exercise, representing an unexpected high level close to maximal exertion of subjects which would correspond to a RER of 1.15. One primary reason for the differences in RER in relation to VO_2_ can be the different muscular strain that occurs during a running treadmill test compared to a simulated firefighting test. Firefighting includes many start-and-stop motions similar to game sports. Previous studies showed that the muscular strain during game sports affected metabolic parameters such as RER differently compared to respiratory parameters like VO_2_^34^,^35^. This unusual VO_2_/RER relationship has also been found by Harvey *et al*.^15^ and Williams-Bell *et al*.^18^ during firefighting exercises. Another possible explanation for that can be the influence of a firefighter’s turnout gear and equipment (e.g. SCBA) on different physiological variables. According to Perroni *et al*.^36^, wearing additional weight in terms of full protective clothing and the SCBA reduces a subject’s VO_2peak_ by ~27% from 55 to 43_ _ml/min/kg. Therefore, and based on our findings, we suggest that assessing only mean VO_2_ values from a simulated firefighting test will fail to represent the demands of firefighting. The pattern of RER provides considerable inside into the true metabolic demands which we found were hidden when assessing only mean VO_2_-values.

Mean minute ventilation (67.5 ± 13.2 l/min) across the whole REPE_spirometry_ exercise was lower compared to the values reported from Holmer and Gavhed^27^. Mean breathing frequency was highest averaging 38.9 ± 5.8 breaths per minute during the last task, the *orientation section crawling.* In contrast, subjects mean breathing frequency in the final minute of maximum treadmill testing averaged at 41 ± 5 breaths per minute. Accordingly, subjects were close to their maximum breathing frequency during the *orientation section crawling,* which indicates the strenuous nature of the exercise. These findings are underlined by McArdle, Katch, & Katch^37^ showing that breathing frequency increased to 35 to 45 breaths per minute during strenuous exercise. Subjects consumed 54% of the capacity of a nominal 30 min-cylinder during the REPE_standard_ circuit which lasted 801 s (13.2 minutes). However, these rates of compressed air consumption from the SCBA would have depleted the air supply after 1464 s (24.4 min) and that is before the nominal time of 30 min described for the cylinders. These findings are in line with the data reported from Williams-Bell *et al*.^20^ who determined similar air consumption rates (~51%) and mean VO_2_ values (24 ml/min/kg) for a firefighting exercise of 12.1 minutes duration. For example, the best firefighter regarding his TSA-Score (−4.81) completed the exercise in 10 minutes, consumed 90 bar of compressed air while showing a mean heart rate of 74.2% HR_max_. He had a VO_2peak_ -level of 49 ml/min/kg, spent 72% of time in zone 1 and breathing frequency averaged at 19 breaths per minute. With the shown values he would not have depleted the air supply before the nominal time.

The reasons for the selection of time for completion, heart rate and air depletion rates for our firefighting performance formula were primarily based on the rationale nature of firefighting. When arriving at an emergency scene, firefighters have to work as fast as possible in order to e.g. save lives or prevent the spread of fires. Furthermore, previous data showed that less fit firefighters experienced higher physiological strain near HR maximum not being able to sustain operating speed and therefore not being able to complete firefighting tasks successfully^12^. Finally, firefighters can run out of air supply due to the limited amount of air compressed in the SCBA. Based on these considerations each of the three factors is thought to be important so that all three parameters were included into our TSA firefighting performance formula. Since at present we do not know which factor is most important or whether one factor is more important than another, we used z-transformations in order to avoid unintended weighting of one of the three parameters.

The results demonstrate that performers of different TSA-levels showed significant differences in maximal endurance parameters, the capacity to work below their ventilatory threshold 1 and breathing variables. This is a strong argument that a high firefighting performance comes along with a good aerobic metabolism and confirms our initial hypothesis: firefighters with lower air depletion from the self-containing breathing apparatus, faster completion time and lower physical strain during the simulated firefighting exercise possess a higher aerobic fitness level in terms of VO_2peak_. Indeed, this can clearly be proven by a low TSA-score which can be seen as evidence for the usefulness of the new developed firefighting performance formula. For practical application, the TSA-formula also can be used without z-transforming all three parameters. The product of time for exercise completion, mean heart rate and air depletion from the SCBA (Time*Strain*Air Depletion) is highly related to our z-transformed TSA-formula (Time + Strain + Air Depletion) (*r*_*s*_ = 0.974, *p* = 0.000). However, further research is needed to validate the model, as a variety of weighting options may even improve predictability of firefighting performance.

Out of all parameters we measured we identified the most important firefighting determinants by means of multiple regression. We found a combination of laboratory (VO_2peak_) and occupation-specific parameters (breathing frequency and time spent in intensity zone 1 during the simulated firefighting exercise) that predicted TSA-score best, accounting for 70% of the observed variance. These results are in line with the results of Sothmann *et al*.^12^, who were one of few research groups recommending a subset of screening tests for firefighters incorporating simulated work tasks as well as fitness measurements. In their study the combination of test items such as hose drag, high rise pack, arm cranking and lifting accounted for 50% of the variance associated with the completion of a work task circuit. However, like other previous studies, Sothmann *et al*.^12^ only focused on the completion time of the simulated firefighting tasks for the classification of performance. Other authors found combinations of aerobic fitness and strength parameters accounting for variances around 60%^2^,^9^,^14^ and 70%^13^. As one of the main differences between our study and previous research, we could not identify any strength parameters as important performance predictors in the regression model. This is surprising given that the characteristics of typical firefighting tasks such as chopping, carrying heavy equipment require the intense use of upper body muscular strength. Except push-ups (*p* = 0*.039, r*_*s*_ = −0.40), we found no significant correlations between TSA-score and muscular strength, flexibility and balance variables. Moreover, integrating push-ups into the regression model could not increase the predictive power of 70% of explained total variance. However, with regard to the strength fitness profile our subjects showed a comparable strength level to other studies^11^,^14^,^16^,^38^.

Although muscular strength and flexibility in this study did not show significant relevance for the predictive power of job demands, both should be essential components of firefighting training in order to decrease the risk of job injuries.

The results of multiple regression support the idea that aerobic fitness, in terms of VO_2peak_ and the time spent in zone 1, considerably contributes to how fast (time) and effective (low air depletion from SCBA and minimal physical strain) a firefighter can perform his tasks. Treadmill determined VO_2peak_ has been established previously to be important for firefighters^1^,^5^,^9^,^10^,^28^ and thus a high level of VO_2peak_ is postulated. Recommendations for a minimum relative VO_2peak_ - threshold varied between the suggestions of O’ Connell^6^ with 39 ml*kg^−1^*min^−1^ and the recommended values by Gledhill and Jamnik^10^ with 45 ml*kg^−1^*min^−1^. Our results also emphasized the importance of VO_2peak_, as a high VO_2peak_ is related to a faster operating speed, lower strain and lower air depletion from SCBA. Based on our results, we now recommend a slightly higher minimum VO_2peak_ of 46 ml*kg^−1^*min^−1^ as this value was identified for subjects showing at least average performance in terms of TSA-scores.

In addition to VO_2peak_ as one of the primary determinants of aerobic endurance performance^30^, the time spent in zone 1 was identified to be the second most important fitness factor. Therefore, heart rate kinetics and the contribution of aerobic energy sources need to be considered to play a major role in preparation and shaping of fit and healthy firemen. Furthermore, we found a strong correlation (*r* = 0.69, *p* = 0.001) between VO_2_ at VT1 on the treadmill and the time spent in Zone 1 across the REPE exercise. This means that subjects with a high percentage of time spent in zone 1 possessed a high VO_2_ at VT1. Those subjects can work at a higher exercise intensity while still covering the energy demand aerobically. Lemon and Hermiston^28^ pointed out that firefighters with a higher VO_2peak_ and a high VT1 (as %VO_2peak_) are able to supply a greater percentage of the total energy demand aerobically which results in more work efficiency in terms of total physiological demands on the organism. These findings can help to design endurance exercise programs for firefighters more detailed by focusing not only on VO_2peak_-training but also improving VT1. According to Jones and Carter^30^, an improvement in VT1 with training is a clear marker of an enhanced endurance capacity.

In our study, VT1 averaged at 49% of VO_2peak_ across all subjects. This is a strong indication to extend basic aerobic endurance training, as values between 50 to 60%VO_2peak_ are related to a low basic endurance level^39^. As suggested by Farrell *et al*.^40^, aerobically better trained subjects can exercise at 75–85% of VO_2peak_ while still covering their energy demands aerobically. We therefore recommend a VT1 at 60–80% VO_2peak_ for professional firefighters as it would allow better metabolic adaptation to physical work at this level^41^. It would also enable to increase Zone 1-fraction and reduce the physical strain during firefighting, respectively. Furthermore, breathing rate can be sustained at lower intensity levels and the blow off of the extra CO_2_ produced by the buffering of lactic acid metabolites is reduced. Oxygen needs can then be primarily met by an increase in tidal volume instead of increased breathing frequency. Moreover, increased breathing frequency was identified to have a negative effect on TSA-score based on the results of multiple regression.

## Conclusions

Firefighting is a physically demanding activity challenging both the aerobic and anaerobic system. While other studies researching firefighting activity focused on VO_2_ and HR, we strongly emphasize to also take RER values and VT1 into account when assessing the fitness level of firemen. Based on the results of our study, we recommend a 3-fold fitness analyses for firefighters that allows for designing optimized, detailed and individualized exercise programs for firefighters:

1. Conducting a maximum treadmill test to determine VO_2peak_, VT1 and RCP
2. Conducting a simulated firefighting exercise to determine physical strain with the help of three physiological intensity zones
3. Using our new developed model TSA: *HR*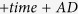 to characterize performance during specific firefighting simulation.

This approach will help to improve firefighters’ physical fitness in order to work healthy, safe and effective. For practical application, the TSA-formula also works without z-transformations and can therefore serve as a simple model for daily use in fire brigades.

## Additional Information

**How to cite this article:** Windisch, S. *et al*. Relationships between strength and endurance parameters and air depletion rates in professional firefighters. *Sci. Rep.* **7**, 44590; doi: 10.1038/srep44590 (2017).

**Publisher's note:** Springer Nature remains neutral with regard to jurisdictional claims in published maps and institutional affiliations.

## Figures and Tables

**Table 1:** Description of balance, flexibility, strength and muscular endurance testing.

Test	Description
*One-leg standing with eyes closed*	Subjects had to show their one-leg standing balance with eyes closed for 15 seconds. Subjects stand on the supporting leg with the free leg raised and bent 90°. Assessment scheme: 2 points – Subject remained unmoving for 15 s. 1 point – Subject performed the exercise with compensation movements. 0 points – The supporting leg leaves its position (jumping as compensation movement) or the free leg was moved back to the ground or eyes were opened.
*Sit and Reach Testing*	This test, performed on a traditional 32.4-cm-high and 53.3-cm-long box, was used to obtain flexibility assessments for lower back and hamstring muscles. The subject sat on the floor with its legs fully extended with the bottom of the bare feet against the box. The subject placed one hand on top of the other, slowly bended forward and reached along the top of the ruler as far as possible holding the stretch for two seconds. The distance reached by the subject’s finger tips (cm) was recorded. The test was performed three times. The average of the three distances were calculated
*Standing Long Jump*	Subjects placed their toes behind the takeoff line and were instructed to jump as far as possible forward with arm swing being allowed. The best jumping distance (cm) out of three trials was registered
*1-RM testing legpress (one-legged*)	Maximum strength was obtained using a predictive one-repetition (1-RM) formula as described by Brzycki^42^. Values (in kg) were taken from the left and the right leg and the maximum values of both legs averaged
*Hand grip strength*	The grip size of the force dynamometer (Jamar: Lafayette Instrument, Lafayette USA) was individually adjusted to fit the proximal interphalangeal joint of the third finger. In a standing position, with the elbow bent 90 degrees alongside the body, the subjects squeezed the dynamometer as hard as they could. The best of three trials on each hand was registered for the maximum (in kg) and the maximum values of both sides averaged
*Push-ups (reps to fatigue*)	The test was taken as the number of times the firefighter could perform push-ups with shoulder-width space between his hands at a rate of 30 lifts per minute
*Partial Curl-Ups*	The partial curl-ups test was used to measure muscular endurance of the abdominal muscle^43^. The total number of properly performed curl-ups (reps to fatigue) was recorded.
*Shoulder Press (20* *kg*)	In a sitting upright position, subjects grasped two 10 kg dumbbells with a pronated grip, vertically pushed the attachment from chin level up to straight arms overhead, and then pulled back to the starting position. The test was taken as the number of times the firefighter could raise and lower the dumbbells in a seating position at a rate of 25 lifts per minute (metronome set at 50)
*Rowing*	In a seating position, the subjects grasped two 7.5 kg dumbbells with a pronated grip. The test was taken as the number of times the firefighter could row in a seating position by abducting arms (90°) in sagittal plane with two 10 kg dumbbells. The weight was lifted between the spina iliaca anterior superior and the chin at a rate of 30 full lifts per minute (metronome set at 60). The number of completed lifts was registered

**Table 2:** Aerobic fitness, muscular strength, flexibility and balance testing.

Variable	Mean	±SD
Treadmill total time to exhaustion (min)	10.5	1.2
VO_2peak_ relative (ml/min/kg)	45.0	6.0
VO_2peak_ absolute (l/min)	3.75	0.43
HF_max_ (bpm)	181.2	11.1
V_E_ at VO_2peak_(l/min)	126.5	29.4
Leg press (one leg) kg	125.5	31.6
Hand grip (kg)	58.7	7.1
Partial-Curl Ups (reps to fatigue)	82	34
Push-Ups (reps to fatigue)	29	16
Shoulderpress (reps to fatigue)	23	6
Rowing (reps to fatigue)	10.1	3.0
Standing Long Jump (cm)	219	22
One-Leg Standing Score (eyes closed)	1.1	0.5
Sit and Reach (cm)	9.3	3.3

Data are means ± SD. BMI: Body Mass Index, VO_2peak_: peak oxygen uptake during maximal treadmill running, HF_max_: peak heart rate during maximal treadmill running, V_E_: Ventilation.

**Table 3:** Multiple regression model (using Enter-Method) to predict optimal firefighting performance (TSA-score).

Variable	β	Standard error β	β-weight
Model			
Relative VO_2max_	−0.193	0.038	−0.508
Time spent in Zone 1	−0.037	0.010	−0.389
Mean breathing frequency (BF) during REPE_spirometry_	0.122	0.043	0.256

Multiple r = 0.850, r^2^ = 0.723, corrected r^2^ = 0.701, standard error = 1.250.

**Table 4:** Characteristics of the firefighters with TSA-scores ranked into 5 categories: *“Outstanding”* (TSA < −2), *“Above Average”* (TSA −1 to −2), *“Average”* (TSA −0.99 to +0.99), *“Below Average”* (TSA 1 to 2), *“Poor”* (TSA > 2).

		REPE_standard_Time (seconds)	REPE_standard_ HR(%HRmax)	REPE_standard_ AD(bar)	VO_2peak_(ml/min/kg)	Zone 1 (% time)	BF (reps/min)	Zone 2 (% time)	Zone 3 (% time)	BORG
Outstanding	n = 6	664 ± 51	71.7 ± 0.04	126.7 ± 19.7	50.8 ± 4.4	55.8 ± 23.1	32.2 ± 7.5	44.2 ± 25	0.0 ± 0	12.0 ± 3
Above Average	n = 9	757 ± 96	75.8 ± 0.05	147.2 ± 16.2	48.2 ± 3.9	37.0 ± 22.7	32.7 ± 5.2	62.4 ± 27	0.6 ± 2	12.0 ± 3
Average	n = 13	799 ± 127	79.7 ± 0.06	157.3 ± 13.6	45.9 ± 4.7	19.0 ± 14.2	34.3 ± 3.5	68.9 ± 28	12.1 ± 21	12.0 ± 2
Below Average	n = 6	830 ± 34	84.5 ± 0.04	178.3 ± 4.1	39.0 ± 5.3	4.1 ± 4.3	34.4 ± 5.5	87.4 ± 7	8.5 ± 7	14.1 ± 1
Poor	n = 7	955 ± 117	84.7 ± 0.04	204.3 ± 21.5	40.4 ± 4.1	3.6 ± 8.8	36.8 ± 2.5	75.6 ± 22	20.8 ± 27	14.3 ± 2

Means ± SD are presented for REPE (Respiratory Protection Exercise) in terms of exercise completion time, heart rate (HR) and air depletion (AD), the three main performance predictors identified by regression - peak oxygen uptake (VO_2peak_), physiological intensity zone 1 (Zone 1) and breathing frequency (BF) as well as additional parameters: physiological intensity zones (Zone 2, Zone 3), ratings of perceived exertion (BORG-scale).
